# Transvaginal strangulated bowel evisceration through uterine perforation due to unsafe abortion: a case report and literature review

**DOI:** 10.1186/s12905-021-01247-y

**Published:** 2021-03-05

**Authors:** Landry Wakheu Tchuenkam, Aimé Noula Mbonda, Joel Noutakdie Tochie, Patrick P. Mbem-Ngos, Hugues G. Noah-Ndzie, Guy Aristide Bang

**Affiliations:** 1 Faculty of Medicine and Biomedical Sciences, University of Yaounde I, Yaounde, Cameroon; 2 Emergency Department, Batouri District Hospital, Batouri, Cameroon; 3 Department of Surgery, Faculty of Medicine and Biomedical Sciences, University of Yaounde I, Yaounde, Cameroon

**Keywords:** Complications of abortion, Uterine perforation, Bowel evisceration

## Abstract

**Background:**

Induced abortion, whether therapeutic or elective, is a surgical procedure frequently practiced worldwide. It is a significant cause of maternal morbidity and mortality. When the procedure is performed in precarious conditions, by unqualified personnel, it leads to serious consequences, including uterine perforation and its associated lesions. Its management remains a medico-surgical emergency.

**Case presentation:**

We present two cases of unsafe abortions performed by cervical dilatation and intrauterine curettage which resulted in uterine perforation and intestinal evisceration through the vagina leading to acute intestinal obstruction. Both patients underwent intensive resuscitation followed by an emergency laparotomy. The first case was a 26-year-old woman living in rural Cameroon. Following a procedure of termination of her pregnancy, the patient noted the presence of bowel at the vaginal introitus associated with signs of intestinal obstruction. She was transferred to a specialized center was after 4 days later of the onset of the evisceration. Considering the gangrened eviscerated terminal ileum, a right hemicolectomy with anastomosis was performed, as well as a suture of the uterine perforation. The second patient was an 18-year-old African living as a refugee in Cameroon. She was referred for abdominal pain in the context of intestinal obstruction with a viable jejunal loop extruding through the vagina. A simple jejunal resection was performed with end-to-end anastomosis and suture of the uterine perforation. In both cases, the postoperative course was uneventful.

**Conclusions:**

Uterine perforation is a serious complication of intrauterine gynecological procedures and instrumental abortion in particular. It can lead to evisceration of the intra-abdominal viscera through the uterine perforation. It is therefore a real surgical emergency with multiple and fatal consequences.

## Background

Abortion is a serious public health issue and a potentially lethal condition in pregnancy. It is defined by WHO as the complete expulsion of the products of conception from the uterus before 20 weeks of gestation or in the absence of accurate dating from the date of onset of last menses as the delivery of a fetus weighing less than 500 g [1]. However, this definition has been adopted in most countries according to their degree of development. For instance, in Cameroon, abortion is defined as the termination of a pregnancy before 28 weeks of gestation or the delivery of a fetus weighing less than 900 g [2].

Abortion and its complications are a significant cause of maternal mortality worldwide, more particularly in sub-Saharan Africa [3]. Indeed, abortion accounts for 8% of the causes of maternal mortality in the world [4], also 99.5% of these deaths take place in low-income countries [3, 4]. Abortion can be spontaneous or induced [5] when triggered by artificial means, for therapeutic reasons or following the woman’s request, without any medical indication (elective abortion) [6]. Induced abortion remains a sensitive and controversial subject in the world and particularly in sub-Saharan Africa because of its moral, religious, socio-cultural and legal components. It is estimated that 52.7 million induced abortions were performed each year worldwide, between 2010 and 2014 [7], hence, an incidence of 35 abortions per 1000 women of reproductive age; this rate is higher in low-income countries (37 per 1000) than in developed countries (27/1000) [7].

When abortion is performed in a health facility having the necessary skilled human resources and equipment it is termed a safe abortion. On the other hand, unsafe abortion sometimes called clandestine abortion is a procedure for terminating an unintended pregnancy performed by people who do not have the necessary training or in an environment that does not meet minimum medical standards, or both [8]. As a result, unsafe abortion is considered to be an independent factor in maternal mortality [9]; Indeed, the risks of serious maternal complications or death are higher during and after unsafe abortion compared with safe abortion [5, 9]. That said, nearly half of the world’s induced abortions are considered unsafe [7]. Also, 97% of these unsafe abortions take place in developing countries [7].

Generally, each country has its own rules and practices regarding induced abortion and elective abortion in particular [10]. In Cameroon, elective abortion is an illegal act, harshly condemned by the penal code. As a result, pregnant women with a desire for an elective abortion often resort to unskilled persons in a non-clinical setting lacking the minimal healthcare standard to perform an abortion.

Women opt for an abortion for several reasons [11, 12] such as unintended pregnancies; the lack of human support and comfort from their spouses, intimate partner and/or family; low socioeconomic level. A pregnancy resulting from rape, the absence of legislation in favor of adequate health service are also determining factors in the choice of remedy [13].

Like any other procedure, induced abortion can cause minor to severe consequences that can be the life-threatening outcomes. This is mainly [5, 9, 14] post abortion hemorrhage, sepsis related to retention of the products of conception and uterine perforation. The latter although rare, can be the cause of serious internal visceral lesions or evisceration. This morbidity and mortality linked to induced abortion are increased when the procedure is carried out clandestinely [9, 15]. In the cases described below, we present two rare and potentially fatal complications of an abortion performed by untrained individuals in a non-medicalized setting. Subsequently, a narrative review was done; the keywords for the literature review were “uterine perforation, evisceration, and bowel”. This study has been reported in line with the “CARE guidelines” [16].

## Case presentation

### Case N° 1

**Fig. 1:**
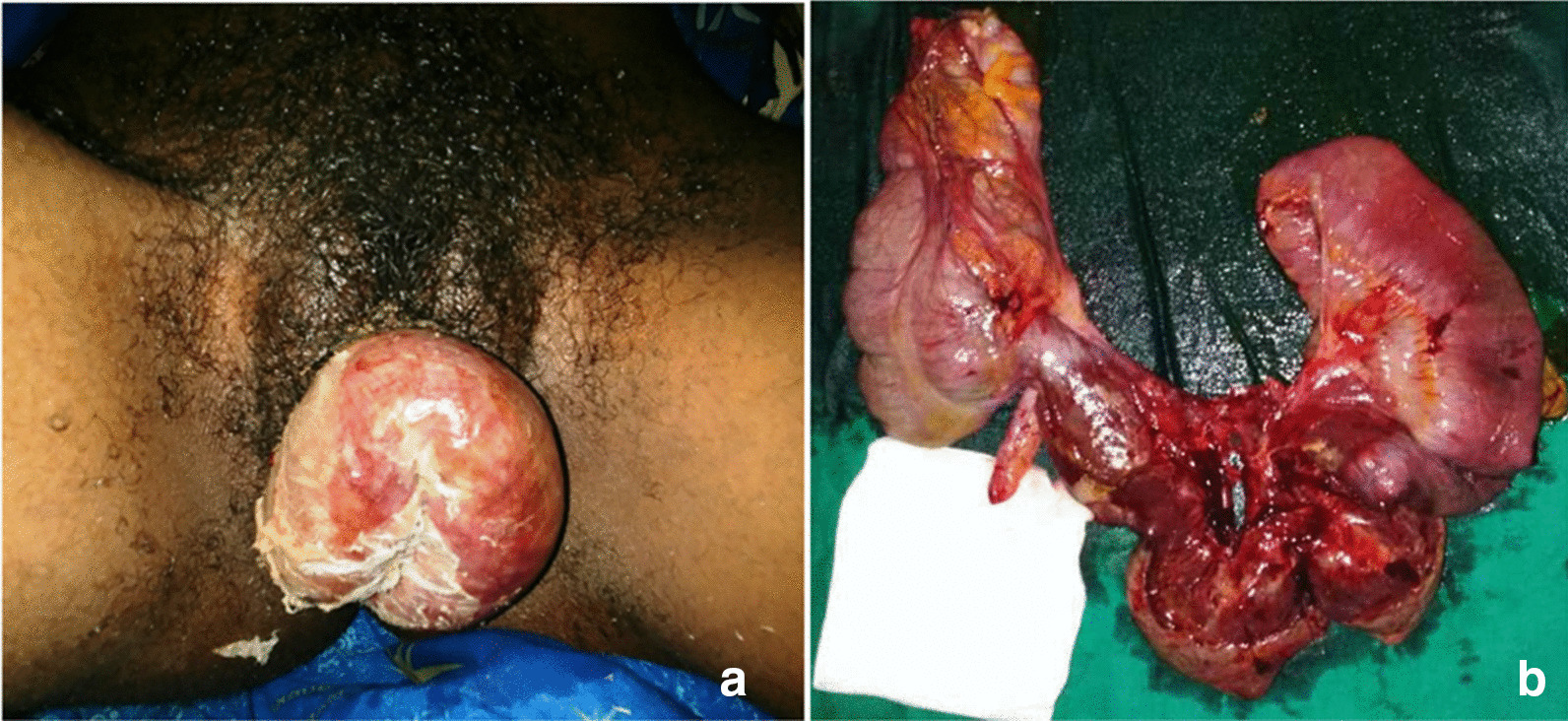
Patient Images_ Case N°1. **a** Transvaginal evisceration, **b** piece of right colectomy

This is the case of a 26-year-old black woman, single, Gravida 3, Para (1) She was referred from a primary health care center located in a rural area to our tertiary hospital for the transvaginal evisceration of bowel through the vagina. Four days before, when she was 10 weeks pregnant she underwent a uterine dilatation and curettage (D and C) performed by a non-certified health care personnel in an infrastructure that was not a health facility and neither equipped for this procedure. After the D and C was carried out she was sent back home. A few hours later a painful protrusion of her bowels out of the vagina till the vulvar region occurred while she was defecating. She immediately sought consult in another primary healthcare facility where she was administered analgesics, antibiotics, and a wet sterile drape was applied to cover the eviscerated bowels. Due to an inadequate technical platform in this center for definitive management she was referred to our tertiary hospital three days later. On arrival, the patient complained of severe, generalized abdominal pain, associated with vomiting and inability to pass stool and gas. Her past medical, family and psychosocial histories were uneventful. On physical examination, the patient was fully conscious and ill-looking. She had signs of severe dehydration. We noted: hypotension at 76/56 mmHg, tachycardia at 122 beats per minute, tachypnea at 32 cycles per minute. The temperature was normal. On examination of the abdomen, there was no abdominal distension, nor tenderness. Examination of the pelvis revealed a protruding loop of gangrenous small bowel through the vagina introitus (Fig. 1a). A laboratory panel requested entailing a complete blood count, protrombin time, activated partial thromboblastin time, serum electrolytes, serum urea and serum creatinine were all normal. Our working diagnosis was acute intestinal obstruction by strangulation of the trans-vaginal evisceration of the small bowel following a uterine perforation secondary to unsafe abortion. Her management consisted of fluid resuscitation through two large bore (G16) intravenous lines, placement of a nasogastric tube for gastric decompression, and urinary catheterization. The vascular filling was done using crystalloids with an improvement in the hemodynamic state. She also received analgesics, as well as an antibiotic combination of intravenous (IV) ceftriaxone and metronidazole. After obtaining the consent of the patient and her family relatives, a median laparotomy was performed within the 6 h hospital admission. The intraoperative findings were as follows: uterine perforation located at the uterine fundus, through which the last ileal loop, necrotic up to the ileo-caecal junction was incarcerated (Fig. 1b). After reduction of evisceration, a right hemicolectomy was performed, followed by a suture of the uterine perforation with vicryl N^o^ (2) The post-operative courses were uneventful. Oral feeding was started on the 1st post-operative day and was well tolerated by the patient. She also received psychological care as well as counseling on the need for contraceptive measures. She voluntarily chose to oral conceptive pills for at least 1 year. Her follow-up till 8 months after the surgery was equally uneventful.

### Case N°2

**Fig. 2:**
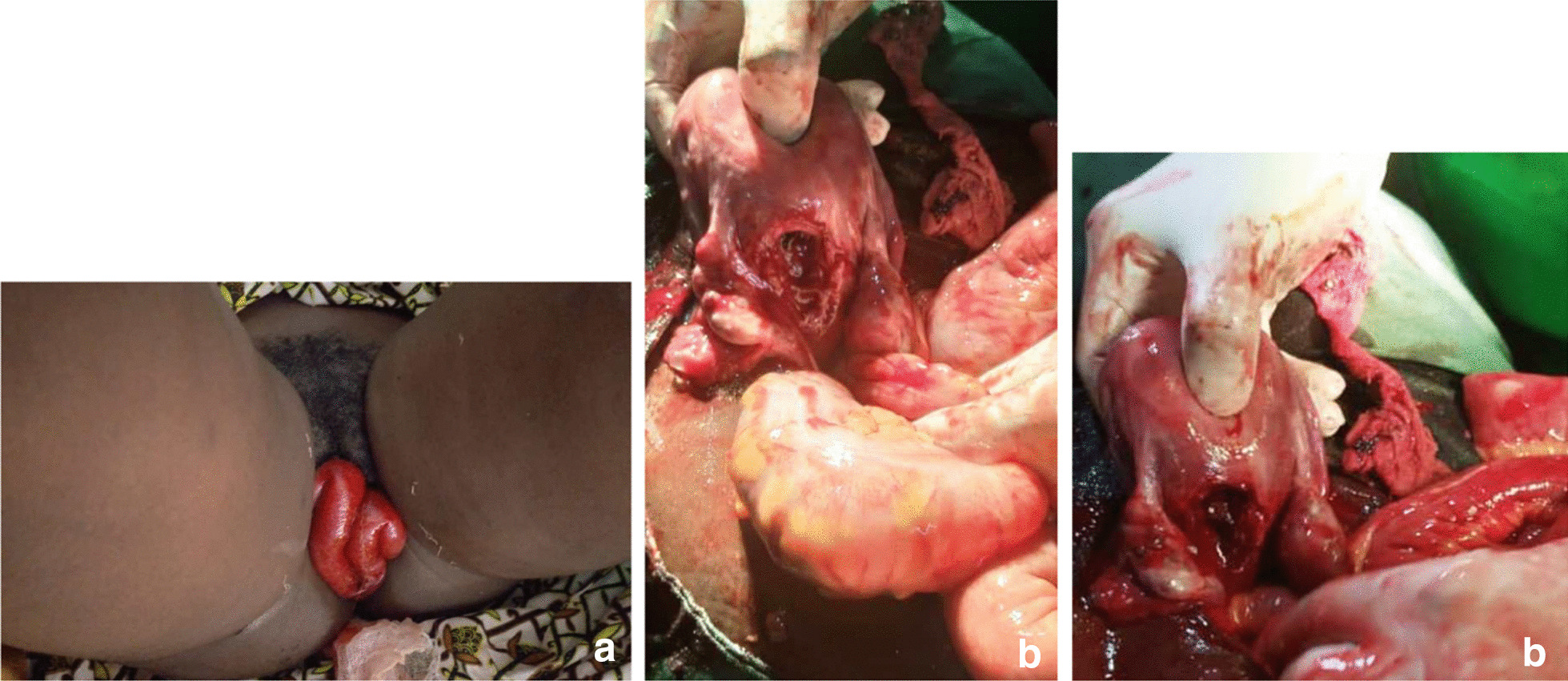
Patient Images_ Case N°2. **a** Transvaginal evisceration, **b** intraoperative view of uterine perforation

The second case is that of an 18-year-old patient Gravida 1 Para 0, a refugee residing in Northern Cameroon. She was admitted for protrusion of intestines out of the vagina that occurred 6 h ago following an unsafe D and C intended for termination of her pregnancy when she was at 12 weeks of gestation. On admission, she had a good general condition. There were signs of acute intestinal obstruction. Hemodynamic parameters were normal, as well as other vital signs. The gynecological examination showed a loop of viable small bowel protruding through the vagina unto the vulva (Fig. 2a). Following a short resuscitation as described above, the patient was operated on by median laparotomy. The findings were a 2 cm diameter uterine perforation located in the posterior part of the uterine corpus (Fig. 2b). Through this perforation, incarceration of the jejunal loop was observed, which was still viable. The surgical procedures were a jejunal resection followed by end-to-end anastomosis, a suture of the uterine perforation and abdominal toileting. The post-operative evolution was normal. Her follow-up till 6 months after the surgery was uneventful.

## Discussion

### Definition, epidemiology, etiology and risk factors

Uterine perforation is a fairly frequent and serious complication of (intrauterine procedure) [17, 18]. It is an uncommon pathology that can be life-threatening as well as compromising a woman’s future fertility [17]. It is defined as a breakdown of the entire full thickness of the uterine wall occurring iatrogenically following a gynecological procedure, usually by a sharp instrument.

The prevalence rate related to the occurrence of uterine perforation is variable depending on the type of intervention performed [17]. Indeed, it is estimated at 5% during the evacuation of retained products of conception for postpartum hemorrhage [17]; 1.6% following a hysteroscopy [19] (more often therapeutic), and at 0.5% after induced or spontaneous termination of pregnancy [14]. Also, partial or complete uterine perforation has been described after the insertion of intrauterine devices [20].

Risk factors have been identified in the literature as predisposing to uterine perforation, we cite uterine anomalies (malposition or anatomical distortion), pregnancy with an increased risk during the second trimester of pregnancy, a uterine scar, even cervical, poor preparation of the uterine cervix before an intrauterine diagnostic or therapeutic procedure and inflammation. However, we identified none of these risk factors in the two cases presented above. Without forgetting procedures carried out by untrained personnel [17, 18]. All these factors contribute to an alteration of the strength of the myometrium wall (body or uterine cervix) which thus becomes conducive to lesions, especially when using a sharp intrauterine instrument [17].

### Clinical manifestations and diagnostic

The clinical manifestations are related to the location of the perforation and the time elapsed since the perforation at the moment of diagnosis. Indeed, a perforation seen late is of poor prognosis compared with one diagnosed early [17]. Most uterine perforations are located on the body of the uterus as seen in the first case above. Other perforations occur at the level of the anterior wall (40%), followed by the cervix 36%, and lastly, the fundus in 13% [18] as observed in case number 2 presented above.

Early diagnosis can be made intraoperatively by direct visualization of the opening or a pelvic viscus (intestines,omentum, or ovaries) through the breach [19]. It can be suspected during the procedure by the loss of resistance during the progression of the instrument, or when the latter progresses beyond the fundal length. It can also be suspected based on signs of visceral or vascular damage, including hemorrhage. The latter can be externalized by the vagina, or be intraperitoneal in the abdominal cavity, or manifest as a hematoma of the broad ligament [21]. The bleeding can be significant when the perforation sits laterally on the uterine body or at the level of the cervix. In these cases, it is reasonable to suspect a lesion of the uterine vessels or one of its branches [18]. In the event of internal bleeding following a perforation that went unremarked during the procedure, the patient may present with progressively increasing abdominal pain, fever or even shock [17].

However, the diagnosis of perforation may be later in the postoperative period. The clinical manifestations are often a persistent vaginal hemorrhage, abdominal pain related to a visceral perforation, hematuria or more rarely evisceration of the abdominal contents, notably the small intestine, the sigmoid, the omentum, and the ovary [22]. The protrusion of the small intestine through a reduced uterine opening (tight uterine opening) leads to incarceration of the loop, particularly serious situation given the complications associated with it [23]. From there, 4 clinicopathological forms can be observed, classified by order of severity [23]: obstruction, strangulation, mesenteric detachment (stripping) and small bowel degloving injury. Therefore, the patient will present with signs of acute intestinal obstruction, or even peritonitis as part of a diastatic perforation. Examination of the pelvis shows an exteriorized bowel loop protruding unto the vulva and perineum; the bowel is dilated and difficult to reintroduce [23]. After 6 h, a necrotic appearance of the loop can be observed, as described in case No. 1. In the case of mesenteric stripping, the intestine is exteriorized in the form of a long tube, non-dilated, with no mesentery observed [23].

### Management

Treatment of uterine perforation can be conservative or surgical depending on clinical manifestations (bleeding) and the risk of damage to the abdominal viscera [17, 18]. The indications for conservative treatment are: asymptomatic patient and perforation secondary to the use of a blunt instrument without an electrosurgical energy source, such as dilators, curette with no suction and hysteroscope. The conservative treatment of the latter indication consists of the placement of a urinary catheter, antibiotic therapy and monitoring in the hospital for signs of bleeding (abdominal pain, abdominal distention, hematocrite and hemoglobin levels), peritonitis or intestinal obstruction.

Surgical exploration is indicated in case of persistent and severe uterine bleeding, in case of suspicion of visceral or vascular lesions, in case of the use of sharp instruments, of suction and in case of perforation occurring after a termination of pregnancy or retention of conception products [17, 18]. Evisceration through the uterine breach is an absolute indication for surgery. Surgical exploration is done preferably by laparoscopy than by laparotomy. During laparoscopy, careful exploration of the pelvis and abdominal cavity should be performed.

In the case of small perforation, several methods can be used to treat the defect: simple interrupted or continuous suture or use of biological substances. Laparotomy is indicated in case of persistent hemorrhage at laparoscopy despite the hemostatic gesture, a large ligament hematoma or an inadequate technical platform [17]. Regarding transvaginal evisceration associated with uterine perforation, the initial management will consist of resuscitation taking into account the consequences related to occlusion or perforation [23]. In a second step, surgery will allow intestinal resection with anastomosis associated with suturing of the uterine perforation, or even a hysterectomy in the event of significant uterine tearing [23].

#### Outcomes

Several cases of uterine rupture during pregnancy or childbirth have been described following uterine perforation [24]. However, the cause and effect relationship is not established, especially since these patients had other risk factors for uterine rupture. This risk must be discussed with the patient following management.

#### Prevention of uterine perforation

The case of safe abortions, prevention measures consist of a preoperative clinical evaluation and preventive measures during gynecological procedures [17, 18]. This preoperative preparation also includes a rigorous clinical evaluation of the patient looking for risk factors for perforation, a correct calculation of gestational age in order to adapt the method of termination of pregnancy and adequate preparation of the uterine cervix [18]. The latter involves progressive dilation using misoprostol, osmotic and/or candle dilators. During the intervention, the additional preventive measures require to position the patient and the uterus adequately and safe use of operative transcervical instruments.

## Conclusions

Unsafe abortion remains a public health significant concern in low-income countries in particular. It increases the risk of maternal morbidity and death through complications such as uterine perforation with intra-abdominal evisceration unto the perineum; septic shock, peritonitis, and multi-organ dysfunction. The authors wish to draw the attention of rare but potentially fatal complications such as transvaginal evisceration following uterine perforation whose management should involve a multidisciplinary approach and taken as a matter of urgency.

## Data Availability

Data sharing is not applicable to this article as no data sets were generated or analyzed during the current study.

## Abbreviations

°C: Celsius degree
D and C: Dilatation and curettage
G: Gauge
mmHg: Millimeter of mercury
N°: Number
TOP: Termination of pregnancy
